# General Architecture for Development of Virtual Coaches for Healthy Habits Monitoring and Encouragement

**DOI:** 10.3390/s19010108

**Published:** 2018-12-30

**Authors:** Antonio Benítez-Guijarro, Ángel Ruiz-Zafra, Zoraida Callejas, Nuria Medina-Medina, Kawtar Benghazi, Manuel Noguera

**Affiliations:** 1 Department of Languages and Computer Systems, University of Granada, 18071 Granada, Spain; ajbenitez@ugr.es (A.B.-G.); nmedina@ugr.es (N.M.-M.); benghazi@ugr.es (K.B.); mnoguera@ugr.es (M.N.); 2 Department of Computer Engineering, University of Cádiz, 11519 Cádiz, Spain; angel.ruiz@uca.es

**Keywords:** telemonitoring, healthy habits, virtual coaches, systems and software architectures, software architecture evaluation, software development

## Abstract

Good health is the result of a healthy lifestyle, where caring about physical activity and nutrition are key concerns. However, in today’s society, nutritional disorders are becoming increasingly frequent, affecting children, adults, and elderly people, mainly due to limited nutrition knowledge and the lack of a healthy lifestyle. A commonly adopted therapy to these imbalances is to monitor physical activity and daily habits, such as recording exercise or creating custom meal plans to count the amount of macronutrients and micronutrients acquired in each meal. Nowadays, many health tracking applications (HTA) have been developed that, for instance, record energy intake as well as users’ physiological parameters, or measure the physical activity during the day. However, most existing HTA do not have a uniform architectural design on top of which to build other applications and services. In this manuscript, we present system architecture intended to serve as a reference architecture for building HTA solutions. In order to validate the proposed architecture, we performed a preliminary evaluation with 15 well recognized experts in systems and software architecture from different entities around world and who have estimated that our proposal can generate architecture for HTA that is adequate, reliable, secure, modifiable, portable, functional, and with high conceptual integrity. In order to show the applicability of the architecture in different HTA, we developed two telemonitoring systems based on it, targeted to different tasks: nutritional coaching (Food4Living) and physical exercise coaching (TrainME). The purpose was to illustrate the kind of end-user monitoring applications that could be developed.

## 1. Introduction

Healthy aging involves the interaction between genes, environment, and lifestyle factors, particularly good eating habits and regular physical activity. Worldwide, the increase in life span has led to an increase in morbidity and mortality as the result of chronic, lifestyle-influenced diseases. Nutrient deficiency diseases are giving way to energy imbalances, so links between diet and chronic disease are becoming clearer [1].

To be healthy, it is essential to maintain a correct balance of macronutrients and micronutrients, and not exceed the amount that our body needs. A lack of control over nutritional requirements is the main cause of cardiovascular diseases [2]. To combat these health problems, it is necessary to promote healthy habits in daily life. Unfortunately, without specific knowledge about nutrition and other health fields, it is difficult to daily manage and control nutrient intake and exercise needed to be fit and healthy.

Visiting health professionals is an appropriate solution to acquire relevant knowledge for these tasks. However, some difficulties may arise in traditional monitoring through periodic visits to a supervisor, for example, due to mobility problems and difficulties in understanding and carrying out traditional control methods [3,4]. Ubiquity and computing features of wearable and mobile devices soon emerged as a suitable technology to try to address these challenges [5].

For this reason, mobile devices have become commonplace in healthcare settings, leading to a rapid growth in the development of medical software applications resulting in mobile platforms. Besides the provision of a particular functionality, these technical solutions present an underlying system architecture that supports developers and ultimately end-users (healthcare practitioners and supervised users) in the consumption of coordinated value-added services and the supervision of progress toward the acquisition of healthy habits.

In this paper, we present a modular architecture that serves as a basis and guidance to build telemonitoring and e-coaching health tracking applications (HTAs) in order to address the lack of reference software architectures in this field. This architecture provides key elements to support the automation of tasks for supervisors, automatic advice for usual guidelines, frequent questions to the monitored users, and detects the demotivation risk of monitored users [6]. The elements proposed are able to be applied to different types of telemonitoring and e-coaching systems. The ultimate aim is that all applications developed following this architecture have access to the main services and functionalities for telemonitoring and e-coaching.

As part of the contribution and to illustrate prospective instantiations of the proposed architecture, we implemented two specific telemonitoring systems: Food4Living for nutritional coaching and the TrainME for physical exercise coaching. For additional explanatory purposes, we developed the Food4LivingApp, an app for mobile nutritional telemonitoring. With these systems, we show how the components of the proposed architecture can be used as building blocks on top of which to implement health telemonitoring applications within a specific domain.

Food4Living and TrainME were produced in the context of an interdisciplinary research project called AVÍSAME. These two systems have been integrated, resulting in a flexible platform that can be seamlessly combined with other physical training and wellness platforms within the project.

As a final stage of this work, we validated our architecture using a survey to analyze the quality of the solution. The survey consisted of dedicated questions to cover the different software architecture attributes required for an e-coaching telemonitoring software system. To do this, we selected a group of telemonitoring software system experts from different areas to complete this survey. The eligibility criteria for the casting of experts was determined on the basis of their membership in the program committee of the Engineering in Medicine and Biology Society (EMBS) [7], and their well-known pieces of work on systems and software architecture and software architectures evaluation.

The remainder of this paper is organized as follows. Section 2 presents the related work. Section 3 presents in detail the general proposed architecture. Section 4 introduces two systems—Food4Living and TrainME—based on the general proposed architecture. Section 5 describes specifically the implementation of one of them, i.e., Food4Living, explained in Section 4. Section 6 shows the validation process with their results and Section 7 presents the conclusions and future work.

## 2. Architecture Principles Related Work

According to Triboan et al. [8], the key guidelines to achieving a careful monitoring process through the development of software applications are (1) nutritional and physical pre-evaluations, (2) health diagnoses, (3) monitoring and evaluation, and (4) active intervention. Based on these guidelines, we designed an architecture that satisfies for each of these aspects. Our proposal offers a scheme to build applications that allow goals to be specified, and user progress to be recorded and monitored with respect to these goals.

The general architecture and the specific adaptations in the literature are structured in different subsystems for each functionality in the monitoring process of users. A subsystem is an element that works independently within the general architecture. In telemonitoring systems, a subsystem can be the software responsible for monitoring the user, the software used to analyze the monitored data, or the remote server. Although, to the best of our knowledge, no standard reference architecture exists for health telemonitoring systems, there are a series of common elements [9,10]. In particular, Hamdi et al. presented a detailed comparison of the most representative European research projects in health telemonitoring and showed that they converge into a common architecture with the following main components. (1) The user monitoring subsystem includes elements that act as gateways, either specific technology embedded in a demotic ambience (e.g., a medical box) or a smartphone, facilitating data collection and user monitoring, and, in some cases, integrating sensors. (2) The caregivers or practitioner’s subsystem, which usually encompasses user monitoring. (3) A remote server connected to the gateway that deals with user authentication, data management, data processing, and user profile and health record management.

Villarreal et al. [10] distinguished several key features: mobility, security in data storage and transmission, user adaptation, and interoperability. Their architecture is based on different layers and modules, which are responsible for isolating the graphical user interfaces from the applications, with submodules that synchronize and operate the data in a central server.

The references reviewed propose elements for the construction of telemonitoring systems, but these alternatives are not specialized in the domain of health and e-coaching. For this reason, the studies we analyzed contain elements for obtaining information about the monitoring process, but lack the necessary elements (modules) to produce and monitor action plans with users during the monitoring process.

To design our system architecture, we studied different alternatives from existing telemonitoring and e-coaching platforms and architectures [11,12]. There are also studies on reference architectures for healthcare, but they do not consider the support to the components and features mentioned above [13].

Our proposal works similarly to previously studied references [11,12], but adds other subsystems targeted to practitioners (healthcare supervisors) that elaborate new information for the monitored users. We handle information representation in platform-independent languages (i.e., JSON and XML) for interoperability purposes. As such, we explain the expected behavior of the system and enable changing the behavior of the system without having to recode it, e.g., by just changing data artifacts regarding diets and plans. To do this, we referenced other monitoring projects of physiological parameters for sanitary purposes. Projects, such as Technology Integrated Health Management (TIHM) [14] or (inCASA) [15], create data models on the monitored parameters in order to establish alerts, predictions, and plans to perform diagnostics adapted to users. This specific kind of architecture is useful to have a reference in order to extract the basis of each general telemonitoring architecture in the heath field.

Telemonitoring tools usually collect information about the behavior of the monitored users. After collection, the supervising professionals carry out an evaluation to improve the process. Thus, the telemonitoring system collects information from the users that a subsystem (application) of the platform synchronizes, then the professional healthcare provider performs an analysis and produces new output (food plan, workout table, etc.) on the basis of the user’s profile data. This analysis or plan is then synchronized and offered back to the monitored user in order to start the cycle again under new guidelines, but without stopping the monitoring process.

## 3. Architecture for Building Telemonitoring and E-Coaching Platforms

In this section, we present a general architecture that provides the basis for the construction of health telemonitoring and e-coaching systems. With this architectural model, we facilitate the development of new telemonitoring and e-coaching systems for different domains such as nutrition, locomotion, and ergonomics, among others. We abstracted the needs and common elements of different types of e-coaching systems, proposing an architecture that can be instantiated in different domains. Once the elements and basic needs are covered, the architecture we present allows certain components to be extended in order to meet the requirements of each specific domain.

**Figure 1:**
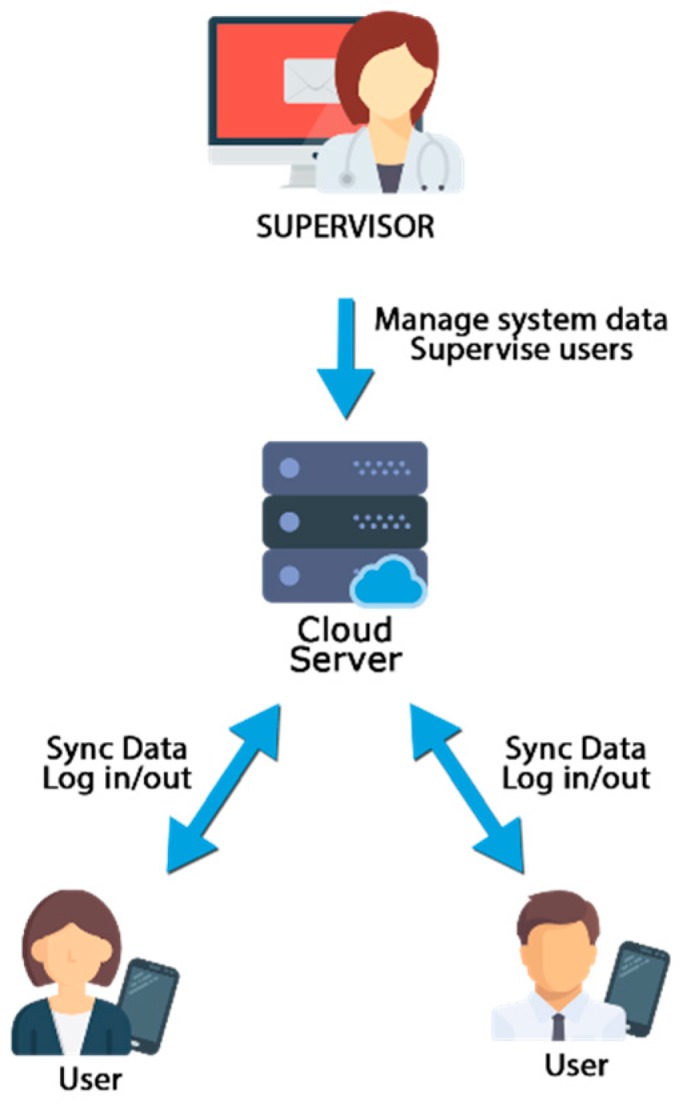
System user overview.

The proposed general architecture is divided into several modules and basic elements to foster the encapsulation of the functionalities and isolate the dependencies between modules. These subsystems will be used by two types of users, supervisors and monitored users, connected through a third subsystem consisting of a cloud server, as shown in Figure 1.

The supervisor subsystem includes a graphical user interface (GUI) to access the different supervision functionalities by supervising users. The mobile user subsystem, for the users that will be monitored, encompasses several modules that interact directly with the user and with other modules, and the cloud storage subsystem that connects all parts.

**Figure 2:**
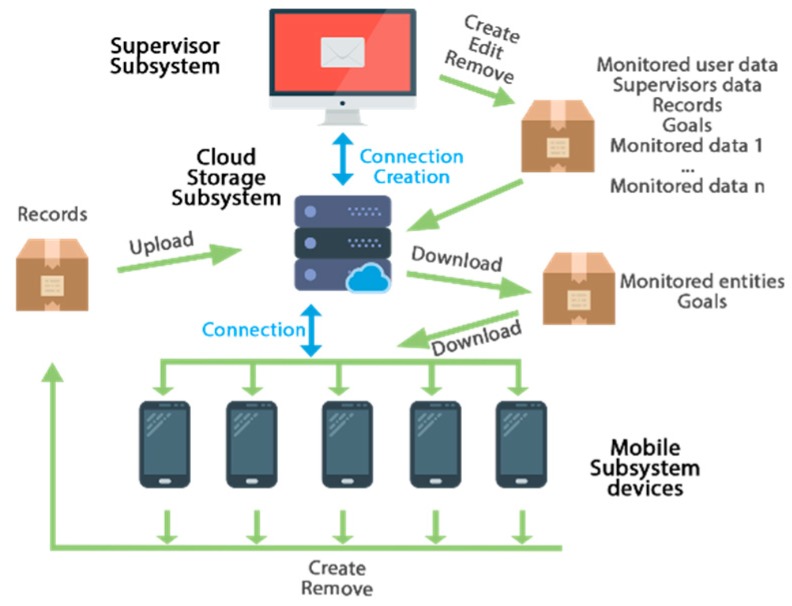
Communication scheme of the system.

The communication scheme between the three subsystems is depicted in Figure 2. The mobile subsystem downloads the available monitored entities needed, such as the user data, the goals models, and other data required for the specific telemonitoring system, whenever there is a new version of the goals or records on the cloud storage subsystem. Similarly, the data of the goals, records, or other aspect about the users will be uploaded or updated in the cloud storage subsystem whenever new records are collected from the mobile subsystem. The supervisor subsystem makes use of all the data in the system (e.g., goals, users, records, etc.) and allows introducing new information through the appropriate administration panel. It also queries the server about information and establishes goals for each user, updating the corresponding data in the cloud storage subsystem.

**Figure 3:**
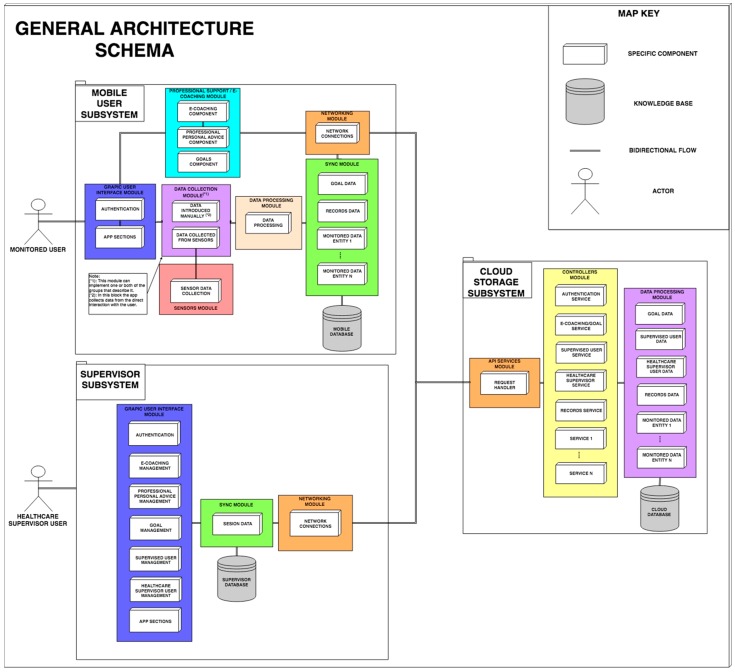
Architecture scheme of the system.

In Figure 3, the different elements of the proposed architecture are displayed (check a more detailed description in the video: https://www.youtube.com/watch?v=haedQb7WySs). These elements are knowledge bases, subsystems, modules, and components. The knowledge base includes the databases necessary for the operation of the platform, which contain information about the monitored items such as nutritional or exercises data, and other information, such as platform user information. They also store information to produce statistics for healthcare supervisors. In addition, we configure the bidirectional flow connector. This symbol indicates the data flow between the different components or modules that are connected. With this symbol, we explain that the data produced on both sides are shared to produce new results.

In Figure 3, we highlight three subsystems that work in parallel and are coordinated: the mobile subsystem for users’ telemonitoring, the supervisor subsystem, and the subsystem in the cloud.

### 3.1. Mobile Subsystem for User Telemonitoring

This subsystem is formed by several modules and components to support different functionalities. These include a GUI module that offers a friendly interface, a module for user authentication (Authentication), and a module responsible for the main functionalities that will be shown to the users (App sections).

Another important module, the Professional support/e-coaching module, offers the user personalized support from their supervisor and incorporates mechanisms to provide automated guidance. This is a key state-of-the-art module in the proposed architecture, so it is described in more detail in Section 3.1.

Another relevant part of the mobile subsystem is the Data Collection Module. This module obtains from the user data to be monitored. This component may consist of one or two subcomponents for automatic data collection from sensors and/or explicit data recording, respectively. These subcomponents are data collected from sensors and data introduced manually. Data collected from sensors serves as an adapter and processor of the information obtained through active sensors. It includes a layer (sensor data collection) for interpretation and reception of the information extracted from heterogeneous sensors (e.g., Zephyr BioHarness 3.0 [16,17]). As such, the coach may integrate different third-party services for capturing data from sensors, such as external APIs of products such as FitBit, Google Fit, etc. The data introduced manually subcomponent serves as a mechanism for obtaining data manually not obtained from sensors, through the direct interaction of the user with a mobile subsystem interface (data introduced manually).

Another part in this subsystem is the Data Processing Module. This module, based on the data obtained, performs processing and/or adaptation of the data, producing an interpretable result for the professionals who supervise the user. The Synchronization Module stores, collects, and synchronizes the information externally with the cloud subsystem that is monitored or requested by the monitoring system. The Synchronization Module is composed of each of the controllers that handle all the interactions with local offline storage of the monitored entities data (data of the monitored entity).

The Network Connection Module allows the mobile subsystem to make the necessary connections with the cloud to synchronize the data (Network Module). Finally, the Mobile Subsystem uses the Mobile Database to store the subsystem data.

### 3.2. Supervisor Subsystem

The Supervisor Subsystem is used by health professionals to monitor and design actions for the monitored users and is formed by several modules and components with different responsibilities. A module (Graphical User Interface (GUI), in our case) that works similarly to the GUI module from the mobile subsystem previously explained. In this case, the GUI module serves the graphical interfaces to the supervisors to perform the analysis of their supervised users (App sections).

A Network Connection Module allows the supervisor subsystem to make the necessary connections with the cloud to synchronize or obtain the necessary data (Network Module).

### 3.3. Cloud Subsystem

This subsystem serves as an information serving entity, registering, connecting, offering, and processing data from the other subsystems of the monitoring system. To perform these functionalities, the subsystem consists of several components with different responsibilities. A web component collects all the necessary calls offered by the network components of the other subsystems (API). A mechanism responds to the functionalities requested by the calls of the other subsystems, handling the requests and identifying their needs (Controllers), offering a driver for each service provided. An information processing module (Data Processing) collects, saves, manages, and processes data regarding each entity needed in the monitoring system (data of the monitored entity). A storage system of the monitored entities data appears in the subsystems. The mobile applications register offline in a database data about user progress, and periodically synchronize the new records with the cloud server through secure requests (Sync Module), updating the new records on the server, accessible by the supervisors through the administration panel (Supervisors GUI).

Also, the API module is responsible for providing the services and resources of the cloud storage for external requests, authenticating such requests with security tokens, whereas the controllers of the modules are responsible for adapting the information extracted from the databases or requests to execute the responses to the requests or to fill in the views of the user interfaces (GUI).

Thus, our proposal is compliant with the key elements of the health telemonitoring architectures described in Section 2, as it integrates a remote server with user authentication, data management, data processing, user profile, and health record management, with subsystems for the monitored users and supervisors.

### 3.4. Professional Support/E-Coaching Modules

In the architecture proposed, we have a specific module in charge of helping and guiding both the monitored users and the professionals in charge of supervision (Professional Support/e-coaching Module). To identify the basic needs that must be covered in an e-coaching assistant, we analyzed several studies that discussed the negative aspects of the telemonitoring systems available today [18]. These studies highlighted the lack of adaptation of the plans to the monitored users, which decreases motivation and is detrimental for user engagement, causing them to abandon their use.

**Figure 4:**
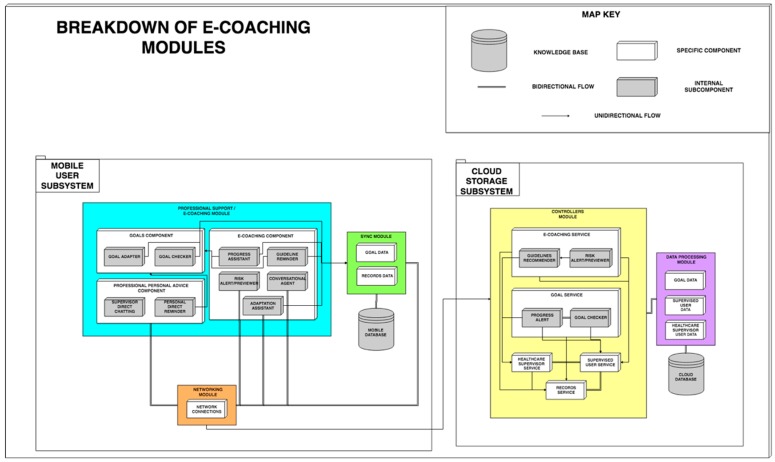
Breakdown of e-coaching modules.

The e-coaching and Professional Support Module is divided into mobile subsystem and cloud storage subsystem, as shown in Figure 4.

Figure 4 highlights a new communication element called ‘unidirectional flow’. This arrow defines the flow direction of the data, which means that the data produced in the origin of the arrow is sent to the destination component or module without another result being returned to the previous element. The module of the mobile subsystem is composed of three components:

- The goal component handles information about the goals of the supervised user, subdivided into two subcomponents: the goal checker, in charge of checking when the goals established by the supervisors are met and the goal adapter, responsible for adapting the goals established on the basis of the progress obtained by the user. For example, if it has been set a nutritional goal of ingesting *X* pieces of fruit per day, with every update about ingestions throughout the day this component would calculate the number of pieces of fruit the user would still have to intake that day in order to attain that goal.
- The professional personal advice component, conceived to create a direct interaction between the monitored users and their supervisors, is composed of two subcomponents: the supervisor direct chat subcomponent, responsible for communicating via messaging, audio, video, or any other method to resolve the pertinent doubts directly between both users and the personal direct reminder subcomponent, through which the supervisor sends alerts to the supervised users to remember goals in progress or to warn them about any issue.
- The e-coaching component is responsible for monitoring the users’ motivation, progress, and adaptation to their goals. This component is subdivided into five subcomponents: (i) the progress assistant subcomponent offers progress feedback to the user meeting their goals, providing congratulations, and offering motivation and guiding to the user; (ii) the guideline reminder establishes reminders about compliance with guidelines/goals; (iii) the risk alert/previewer subcomponent is responsible for analyzing and detecting possible risks of demotivation or adoption of unhealthy habits. To do that, this module analyzes user habits using the app, detecting if the user has stopped recording meals plans, finished the exercise goals, or using the app in general. (iv) The conversational agent is responsible for offering support to solve small common doubts about the adoption of the goals, the usage of the system, or to offer voice interaction between the functionalities of the application and the user. (v) The adaptation assistant subcomponent enables the adaptation of some goals according to the needs or preferences of the user. For example, if the user must reach a certain ingest of calcium and offers the user a dish that they dislike, the adaptation assistant selects another one that the user prefers and meets the goal. In the scope of the physical exercise, it might suggest another type of exercise for the same muscular group.

All these components interact with the Cloud Storage Subsystem through the networking module which makes use of the Synchronization Module (Synchronization Module) of the monitored entities, such as the target data and registered data. The Cloud Storage Subsystem described above contains specific components for e-coaching, user goals, and professional support. These components manage these functionalities directly on the server with

- The e-coaching service, which serves requests made to the API to suggest action plans to the supervising professionals and to rise and manage alerts of risks of demotivation of the monitored users.
  ○ The guidelines reminder supports and automates the process of suggesting action plans to professionals based on decisions made by other professionals in similar situations.
  ○ The risk alert/previewer analyzes and detects routines/guidelines that indicate risky situations, such as demotivation of monitored users, based on a system of rules.
- The goal service, which monitors and alerts about all the information regarding the goals preset to users.
  ○ The progress alert notifies supervisors about the degree of compliance with the goals.
  ○ The goal checker analyzes the goals of the users to establish their degree of compliance.

## 4. Applications of the Proposed General Architecture

In order to demonstrate the versatility of the architecture proposed, we present two specific instantiations of the general architecture outlined in Section 3. These are Food4Living, a specific system for telemonitoring and promoting healthy nutritional habits, and TrainME, another instantiation of the general architecture for telemonitoring and promoting healthy physical exercise habits.

### 4.1. Food4Living: A Cloud-Based Mobile Architecture to Monitor Dietary Habits 

Food4Living is a complete instantiated architecture used to easily develop applications for nutritional telemonitoring and e-coaching based on the general architecture proposed in Section 3.

**Figure 5:**
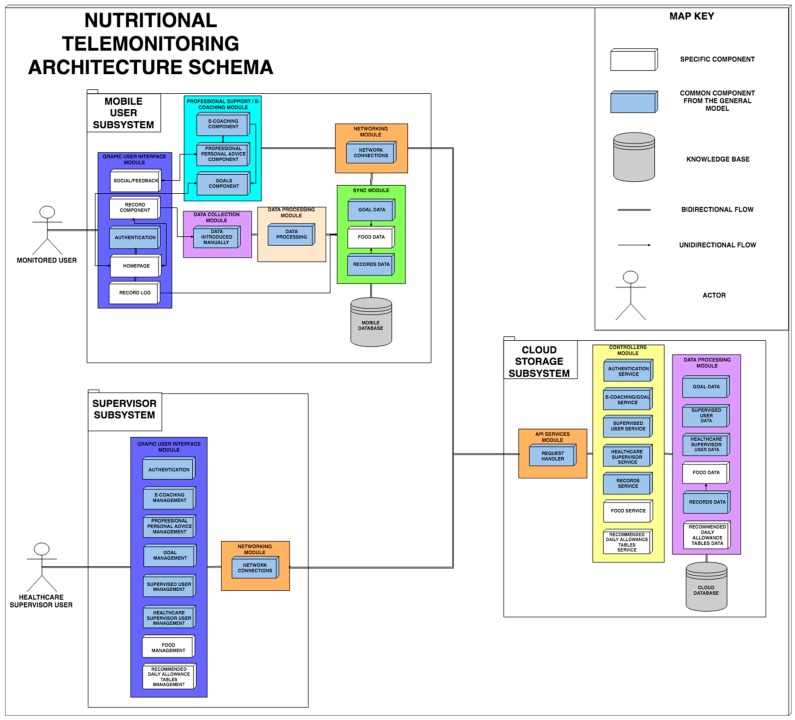
Schema of the architecture for nutritional telemonitoring (Food4Living).

In Food4Living, there are two main groups of users (besides the technical system administrator): (1) monitored users, who employ a mobile application, and (2) monitoring professionals (nutritionists and dietitians) that analyze the relevant data of the monitored users, comparing them with nutritional goal references (recommended daily allowance) (Figure 1). Food4Living is an adaptation of the general architecture explained in the previous section. Food4Living has the same basis (at least the modules from Figure 3) with the common components (white boxes in Figure 3) and new elements that are from the nutritional field. These new elements are highlighted in Figure 5.

The monitored user (hereinafter, user) tracks food records by means of a mobile application. Food4Living synchronizes the user’s data automatically and provides tailored indications and meal plans designed by the nutritionists who supervise the user.

The supervisor nutritionist (hereinafter called the supervisor) uses a panel to collect relevant pieces of data in order to create food plans and nutritional goals adapted to the particular needs and eating habits of the user. Everything is synchronized through a cloud computing subsystem described in the next section.

#### 4.1.1. Food4Living-Adapted Architecture

Food4Living makes use of the common elements of a health telemonitoring system gathered in the proposed general architecture explained in Section 3, and extending other specific components. Figure 5 presents a similar structure as the general architecture, where light blue represents the elements that remained unchanged and the specific components tailored to nutritional coaching domain are in white. The main differences correspond to the specific sections of the graphical interfaces for a nutritional system and in the process of obtaining the parameters to be monitored (as no sensors are used). Similarly, the modules for synchronization and data processing in the cloud specify nutrition entities and controllers for those entities, e.g., food and recommended daily allowance tables.

#### 4.1.2. Key Platform Features

The Food4Living instantiated architecture exhibits the following properties:

- Dynamic data and system adaptability allow the incorporation of new foods with dynamic nutritional information. These are composed of different attributes equivalent to their constituents and nutrients from which different nutritional statistics can be obtained. They can be represented in either XML or JSON (both supported) so that raw aliments can be reused in different meals.
- A key role of nutritional goals is the main communication mechanism between nutritionists and supervised users. By means of goals, nutritionists can establish guidelines and diets adapted to each user. The goals are displayed directly on the mobile platform and are automatically updated with new user reports until the objective is reached.
- System data synchronization. The mobile platform synchronizes the new records and goals when the date of creation/modification is more recent than the one stored in its internal database.
- Multi-user, representing different profiles for administrators, nutritionists, patients and their caregivers. Each profile has different permissions and privileges within the system.
- Interoperability involves communicating information in real time between the three different subsystems of the platform: the user mobile subsystem, the nutritionist subsystem, and the RESTful API (cloud).
- Immediate availability in any environment with or without Internet connection, registering the data in local databases, and synchronizing.

#### 4.1.3. Data Communication in Food4Living

To build an app in Food4Living, the developers must implement only the graphical interfaces (GUI) used to interact with the main functionalities that use the services provided by the RESTful API [19,20] to obtain the data. The nutritionist subsystem was designed according to the model-view-controller pattern [21], with a controller for each important block of requests, such as food management, users, nutritionists, and recommended daily allowance tables. These controllers make use of the REST API to request the necessary data from the database, performing an authentication from a Jason Web Token (JWT) [22]. The data displayed to each user depends on their privileges. This API works as a wrapper between the system information (storage) and third-party applications, offering different types of data according to the routes that are executed in the different services that it manages.

The API is structured into services, which are the controllers responsible for offering the data processed, and differentiating and restricting which requests are available without authentication and which require a valid token. Supervised users will have access to their plans and the possibility to update their tracking. Nutritionists will be able to

- Register in the system the new users to be supervised.
- Create and manage available foods in the system and their nutritional information.
- Manage their own tables of recommended daily allowance.
- Consult and edit the food registries of the users under their supervision.
- Establish and update goals and nutritional plans based on the statistics obtained from their eating habits.

Two of the most important services are the Objective Service and the Records Service, which synchronize the food records of each user with the mobile application and the administration panel of the nutritionist subsystem. The rest of the services of the API serve the information to other modules of the system and to third-party calls.

Likewise, statistics about food intake can be generated in real time and it is possible to generate comparisons, reports, and calendars. The system data are formatted and transmitted to the API in JSON format [23,24]. To secure sensitive pieces of information, responses are encrypted using JWT tokens, providing access to certain parts of the API only if the request is made with a valid access token. All other routes and requests without sensitive data are available through HTTP requests to the API, and receive responses in JSON format.

### 4.2. TrainME: Cloud-Based Mobile Architecture to Monitor Physical Exercise Habits 

TrainME has two main groups of users (besides the technical system administrator): (1) monitored users, who employ a mobile application, and (2) monitoring professionals (personal trainers) who analyze the relevant data of the monitored users, comparing them with objective references (Figure 1).

The user uses the mobile application with the proper sensors to track physical activity records, reflecting their physical activity habits. TrainME synchronizes the user’s data and receives the indications and workout plans from the personal trainers who supervise the user. The monitored user receives notifications and reminders to carry out a complete workout plan. The personal trainer (hereinafter supervisor) uses a panel to collect relevant pieces of data in order to create the workout and exercises goals adapted to the particular needs and performance of the user.

#### 4.2.1. The TrainME Adapted Architecture

Similar to Food4Living, TrainME is a particular instantiation of the architecture proposed in Section 3, inheriting from it the essential elements in each telemonitoring system, and simply expanding other specific components to the physical activity domain.

**Figure 6:**
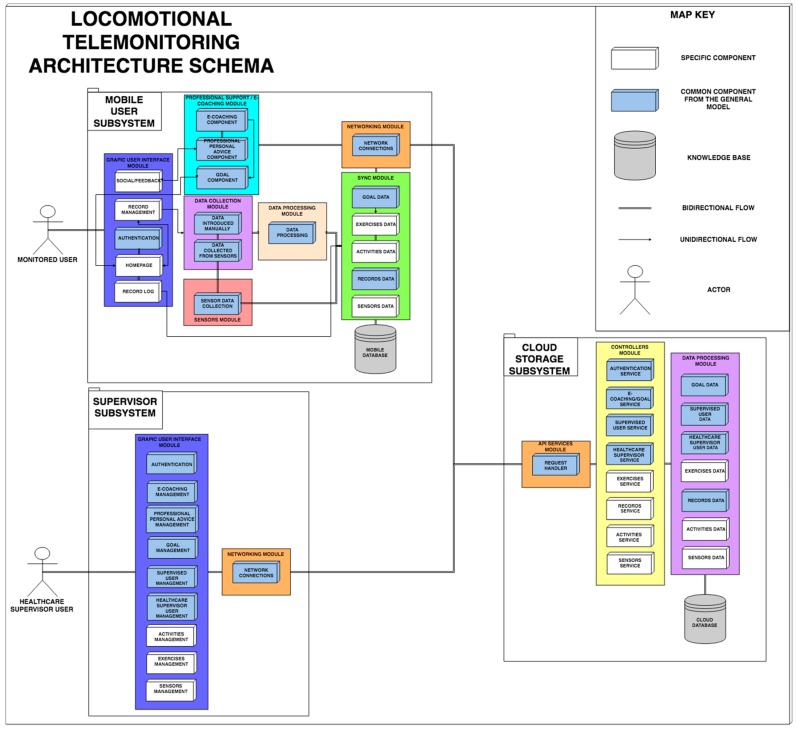
Architecture for locomotion telemonitoring (TrainME).

As shown in Figure 6, TrainME has the same basic structure with the common modules depicted in light blue and the new elements from the locomotion field in white. The differences with respect to the general architecture correspond to the graphical interfaces for a locomotion system, and the process followed to obtain the parameters to be monitored, since, in this case, both subcomponents will be used (data collected automatically from sensors and manually reported exercises). Similarly, the modules corresponding to synchronization and data processing in the cloud have new specific physical activity entities, such as activities, exercises, and sensor data, with their corresponding controllers.

#### 4.2.2. Data Communication in TrainME

The communication scheme works similar to the Food4Living scheme, but with the distinctions corresponding to a different application domain. The mobile subsystem updates (downloads) the personalized workouts and exercises designed by the personal trainer, the data acquired through sensors, the records performed by the user, and exercises goals whenever there is a new version in the cloud server. The personal trainer subsystem uses all the data in the system and the sensor data (e.g., goals, users, exercises, etc.), introducing new information through the chosen administration panel and updating the appropriate data of the cloud server.

To build an app in TrainME, developers must implement only the views (GUI) and consume the services provided by the RESTful API to obtain the data. For the Personal Trainer Subsystem, we followed a design according to the model-view-controller, and used REST APIs and JSON web tokens as explained for Food4Living in Section 4.1.2.

#### 4.2.3. Key Platform Features

The proposed architecture for TrainMe has the same advantages as Food4Living (Section 4.1.3). In this case, the XML or JSON (both supported) representation enables the reuse of elementary movements that can be part of different exercise routines.

## 5. Food4Living Application

In Section 3, we presented the general architecture and in Section 4 we showed it can be successfully applied to create two different specific software systems (Food4Living and TrainME) for heterogeneous e-coaching and telemonitoring domains. As a final verification of the feasibility of the proposed architecture, we present Food4LivingApp, an application developed for Food4Living system and implementing all the guidelines described in this paper.

To explain our proposal, we studied the most popular telemonitoring tools and applications available today for smartphones. Among these, we identified MyFitnessPal [25], Lifesum [26], Diary of Nutrition [27], Freeletics Nutrition [28], and 8fit [29]. All of them are applications through which a user sets goals aimed for weight maintenance, weight loss, and gain muscle mass, and include a food library with food nutrients (macronutrients and micronutrients), to create food records during the day, and also include a workout library with some routines. To support the aims pursued by the users, these applications are designed to set daily caloric plans, create caloric limits depending on the user’s weight objective, and offer weekly progress statistics until reaching the goal. Once the objective of ideal weight is established, the user chooses a deadline, and on the basis of the current weight, height, and level of activity of each user, the application calculates the number of calories to ingest daily.

Many of the most famous applications used to maintain weight and promote healthy habits do not differentiate between each individual, since the proposals or the bases where the caloric limits are established are obtained from general references, not from supervision and personal adaptation by a professional. Additionally, these applications do not have user support systems explaining the guidelines to be followed, and they do not include any supervision or interaction between a professional team and users either, making the guide more complicated in the process of adapting to healthy habits [30].

Unlike state-of-the-art systems, Food4Living allows building applications that are not limited to establishing caloric limits to reach a target weight. Its objective is to allow diets and exercise tables to be adapted to each user based on professional supervision.

One of the benefits of the Food4Living architecture is the possibility of deploying new applications implemented by developers/programmers. As explained previously, we provide an API with well-defined interfaces to enable communication between all parts of the system (API REST). During the design of the API, data format and representation issues were also considered as well as the use of standard (e.g., JSON and XML) technologies to facilitate their extensibility, maintenance, and interoperability with third party applications and systems.

The following applications were developed, supported by a Food4Living system design, a mobile application aimed at supervised users with which to record their nutritional habits, and an application for the nutritionists to manage and supervise the users in their charge.

### 5.1. Security

Security is a key aspect for telemonitoring applications, which is why it was already considered in the proposed architecture and its instances (Section 4.1.2). Thus, security is seamlessly incorporated in the applications that implement them.

The transfer of data between the subsystems is protected by different security mechanisms, using SSL certifications for HTTPS requests, using session encryption through the use of self-generated security tokens with JWT, and maintaining a secure environment with necessary mechanics security for web servers [31]. Finally, the sensitive data of the users are encrypted in the database to protect their content.

### 5.2. Nutritionist Subsystem

Through the nutritionist subsystem, nutritionists register, consult, evaluate, and establish nutritional guidelines and personalized plans for each user under their supervision. To do this, the system orchestrates its different modules by means of the architecture proposed and inserts, processes, and delivers dynamic user data. The system was designed to interpret different data formats, making it scalable and adaptable to new ways of representing the data. As such, it is possible to add new parameters to the food, statistics, and other parts of the system without affecting its structure. The web interface (Graphic User Interface Module) is based on HTML5 and Materialize CSS, and has the following functionalities:

- System of authentication and recognition of different users (Authentication Component): Through the credentials introduced into the system, nutritionists will be able to access the web system through a login form, where the user type (nutritionist or administrator) is automatically recognized.
- General administration panel: The general panel provides access to all the functionalities of the system.
- Nutritionist panel (Supervised User Management Component): Panel showing the list of users associated with the nutritionist, display the main data of the people monitored, and the options to add, display, edit, and eliminate such users.
- User’s personal calendar (Supervised User Management component, Goal Management component, E-coaching Management Component, and Professional Personal Advice Management component): This is the calendar where the supervised users’ food records and goals are displayed. The nutritionist will be able to interact with clicks, eliminating or adding new data to the desired date. This calendar is the main tool of communication with the supervised users; there, nutritionists can observe their daily progress, breaking down the intakes into the different hours of the day, and observing a summary of intake nutritional composition. The nutritionist can interact with the calendar by clicking on the different days, establishing new records or goals that are visible to users from the mobile application. It is also possible to interact with the goals or records already inserted to obtain more details or to remove them from the patient’s calendar. Nutritional goals are diet mechanisms where nutritionists associate a target food to eat, a certain amount, and a deadline to achieve that goal. The goals are updating their progress according to the food records of the monitored user. It is possible to enter daily goals or goals with a certain duration. With this mechanism, nutritionists can create specific diets by setting a specific time for each food in the goal, e.g., by establishing a food goal of drinking a glass of milk at breakfast and establishing the specific day for that goal, continuing with another food for lunch, etc.
- User statistics (Supervised User Management Component, Goal Management Component, E-coaching Management Component, and Professional Personal Advice Management component): This is the section that generates a statistical report of the supervised user from the records recorded in the selected month, comparing these results with a table of recommended daily allowance (RDA) registered by that nutritionist and previously selected and where to observe a weekly summary disaggregated in timetables for the main macronutrients.
- Administrator panel (Healthcare Supervisor User Management Component): This is the management panel for the system administrator to add, edit, or remove nutritionist users.
- Food management (Food Management Component): This panel allows the creation, modification, and elimination of new foods in the system. Such foods will be used by the mobile application and the patient’s personal calendar for the incorporation of new food records or goals. It should be noted that this section indicates the elements necessary for food registration, that is, the photographs to be used in the mobile application, their corresponding portions, and all the nutritional information of the food. The quantity selection system developed is based on photographs that visually represent the portion’s size. For example, when the user reports drinking skimmed cow’s milk, they can choose the image of a small cup, a medium cup, or a large bowl, where each container corresponds to a different portion.
- Management of RDA tables (Daily Allowance Tables Management Component): This is a panel for the creation, modification and elimination of tables of information to make the statistical comparisons of the people monitored and to generate the reports already described above.

### 5.3. Mobile Subsystem

The main tool for the monitored users is an Android-based mobile application where they can record their food intake throughout the day, transparently synchronizing the data, so that the supervisors can keep track of the user almost in real time. Through a simple interface, users can record the food ingested using a form where, with three steps, they select the timing of the intake, the food, and quantity consumed. As described before, we implemented a quantity selection system of images that show representations of different portions.

This system module has the following functionalities.

**Figure 7:**
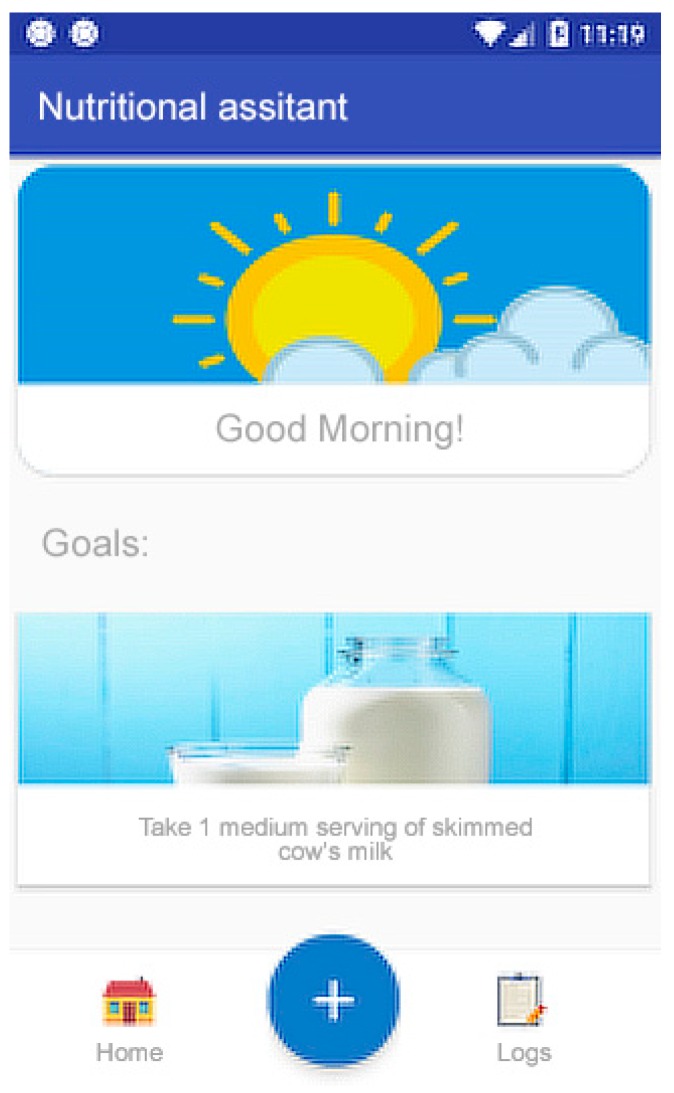
Welcome screen and display of outstanding goals.

**Figure 8:**
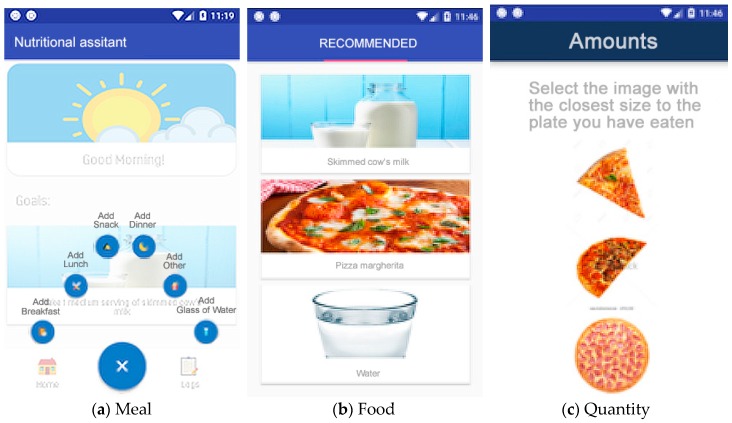
Drop down to register a meal.

**Figure 9:**
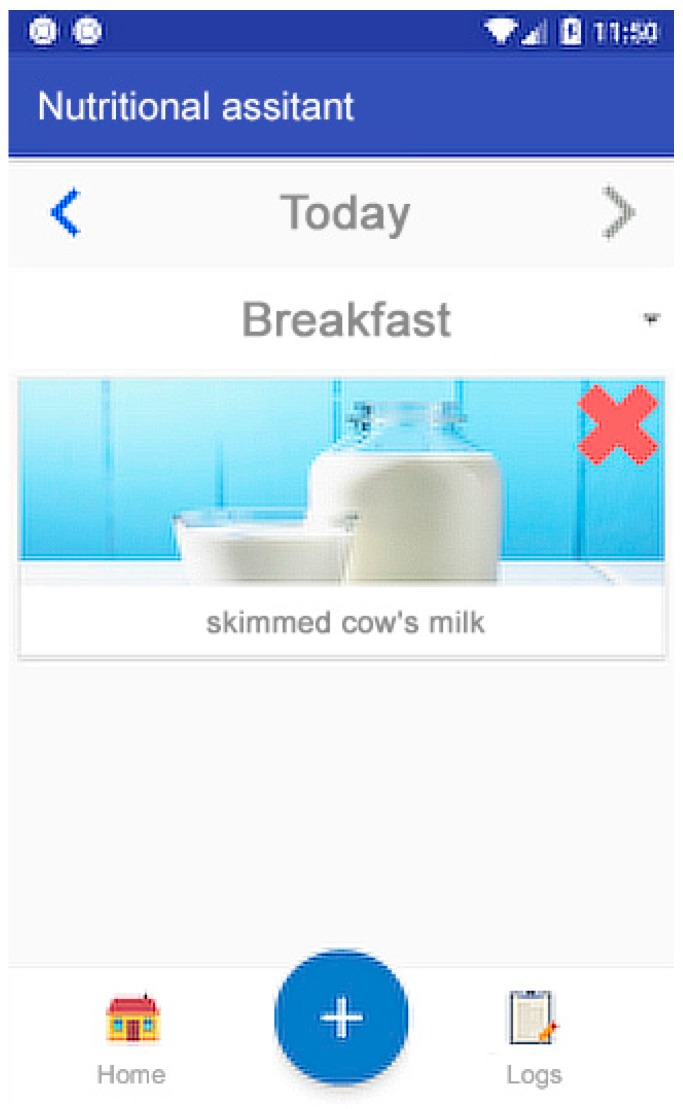
Record display panel.

**Figure 10:**
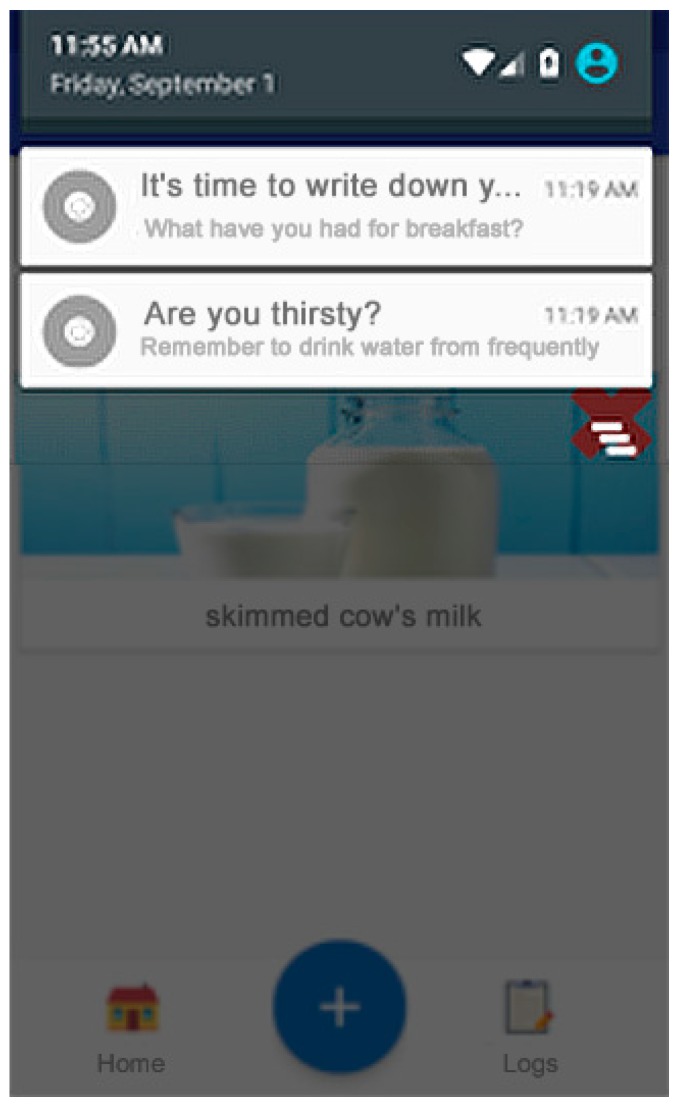
Notification reminder system.

- Secure authentication (Authentication Component): Authentication protected by a generated token that only allows the connection to be opened from a point, thus making it inaccessible to others.
- Welcome screen and display of objectives (Homepage Component, Social/Feedback Component, and Goals Component): An initial screen greets the user and shows interesting information, such as today’s pending goals, indicating the plan for the day (meals and portion sizes). This screen also shows the main buttons of the application (Figure 7). Attending to the usability needs of the elderly, the interface has been simplified to show only the most essential elements: return to the home panel, add a new record, or view the annotated records. The Objectives (goals) panel shows the user’s daily goals that have not been achieved.
- Records system (Record Component Data Collection Module, Data Processing Module, and Record Log Component): First, the nutrition assistant asks about the kind of meal to register (Figure 8a). This food selection screen suggests foods obtained from the local database of the application (Figure 8b). Then, a quantity selection screen (Figure 8c) is displayed using the method described above (Figure 8 and Figure 9).
- Notification reminder (E-coaching Component and Professional Personal Advice Component): When aging, people gradually loose the feeling of thirst, so one of the most important functionalities of our solution is to remind users to drink and eat the appropriate meals during the recommended periods. Through the notification system of mobile devices, the application launches reminders to drink water on a regular basis, and from preset hours, reminds the users that they must report their meal in the application (Figure 10). To do this, different services are used to manage the system of alarms of the operating system in the background.

## 6. Validation Process

In order to verify the validity of the architecture proposed in this paper, we analyzed the properties that a correct architecture must have and asked international experts whether they were covered in our proposal.

### 6.1. Questionnaire

We created a questionnaire covering the main properties identified in Fielding et al. [20] and listed below.

- Performance: Refers to the time required to respond to events or the number of events processed in a fixed time interval.
- Reliability: The ability to maintain the operating system against failures over time.
- Availability: The time that the system is up and running.
- Security: Measure of the system’s capability to resist unauthorized usage.
- Modifiability: Ability to make changes in the system quickly and cost effectively.
- Portability: Aptitude of the system to run under different computing environments.
- Functionality: Capability of the system to meet the established requirements
- Variability: Ability of the architecture to accept changes or extend particularities.
- Subsetability: Capacity to support the development of a subset of the system.
- Conceptual integrity: The property of the architecture that allows unifying the design at all levels. This property shows that the evaluated architecture solves the problem with a reduced number of components without redundancy.

The questionnaire devised (Table 1) contains 10 statements related to each of the properties explained. The respondents were instructed to indicate their agreement on how the proposed architecture satisfied each statement. The agreement level was expressed in a four-point Likert scale with DK/NA option.

We created an online survey that contained a summary of the architecture’s objective, a description of the architecture and its purpose, demographic questions, the questionnaire, open questions on the satisfaction with the architecture, and requested suggestions on how to improve the proposed solution.

### 6.2. Discussion

The survey was completed by 15 experts in software development with at least five years of experience (min. 5, max. 55, avg. 23, and SD 14) in different areas including education, research, and industry. The participants were from different countries, mainly United States of America, Chile, Brazil, and Canada.

To interpret the survey’s results, we analyzed the answers of the 15 participants to the 10 questions, obtaining a total of 150 responses. The answers were measured on a scale of agreement levels between the participant’s professional opinion and how the proposed architecture satisfies the different key aspects. There were five levels: (1) DK/NA; (2) total disagreement; (3) partial disagreement; (4) partial agreement; and (5) total agreement. Table 2 presents the results of the survey.

As shown, the majority of experts agrees with the statements in all cases. The average inter-rater agreement using the Fleiss’ Kappa coefficient [32] was 0.67, which can be interpreted as substantial agreement according to previous reports [33,34,35].

Thus, the results show that the majority of the professionals surveyed agreed with the statements that consider the proposed architecture adequate, reliable, secure, portable, and having high conceptual integrity. The interviewees made minor suggestions that we incorporated in the architecture. Some of these changes concerned the representation of the architecture at lower levels of abstraction, whereas others referred to the incorporation of examples of applications developed under this architecture to verify its purpose.

These results, along with the proof-of-concept presented with the specific Food4Living and TrainME architectures and the practical implementation of the Food4LivingApp, led us to the conclusion that the proposed architecture can be successfully used to help develop new heterogeneous telemonitoring e-coaching systems that fulfill the main desirable properties for these systems that cannot be addressed with state-of-the-art approaches.

## 7. Conclusions and Future Work

Telemonitoring is an increasingly important approach to promote healthy habits, especially for older adults. However, the underlying designs of most HTA available in the literature, as well as commercial applications, do not adhere to the referenced component sets. Reusable architectural designs in this domain that can be used as a reference are lacking. For this reason, we proposed a general architecture for developing applications that satisfy the perspectives of developers while addressing the needs of users and professional caregivers. With it, we facilitate the development of the necessary basic constituents of telemonitoring systems based on three interconnected subsystems: user telemonitoring, supervisor subsystem, and subsystem in the cloud. These three subsystems aim to solve problems not addressed by the applications and models that currently exist.

To demonstrate the feasibility of using the proposed architecture in different application domains with varying requirements, we developed two software systems for nutrition and locomotion monitoring, named Food4Living and TrainME, and described how they could be derived from the general architecture. With these implementations, we show how the different subsystems of the proposed model can be integrated, offering a fully functional application as a final result.

We presented an empirical evaluation of the proposed architecture that shows a high agreement among 15 worldwide experts who consider that our contribution presents relevant properties related to performance, reliability, availability, security, modifiability, portability, functionality, variability, and conceptual integrity.

For the next phases of our research within the AVÍSAME project, pilot studies will be started to test the usability of our developments and its perceived usefulness. Currently, we are evaluating the Food4LivingApp with elderly users and professional nutritionists. In addition, we plan to introduce new mechanisms of artificial intelligence and automatic data mining based on user statistics to suggest alternative dietary plans.

## Figures and Tables

**Table 1:** Validation survey questions.

Number	Question
Q1	The subsystems and modules of the proposed architecture satisfy the needs of health system telemonitoring and e-coaching,
Q2	The modularity of the proposed architecture allows easily making changes in the components that form it without affecting the other components too much,
Q3	The design of the proposed architecture allows the implementation of its subsystems to be independent of the technology used.
Q4	The proposed architecture allows easily including new components.
Q5	The proposed architecture allows interacting with third-party services.
Q6	The subsystems are sufficiently independent and self-contained to allow their integration into other architectures.
Q7	Each of the subsystems, their modules, and their components are pertinent for the operation of the system without including unnecessary redundancies.
Q8	The proposed architecture allows the incorporation of security mechanisms for the protection of accesses and unauthorized uses.
Q9	In case of failure of any of the subsystems, the proposed architecture allow other parts of the system to operate normally through the synchronization of local backup copies.
Q10	The proposed architecture allows distributing the load among its subsystems and provides more computing resources when necessary through a cloud system.

**Table 2:** Survey results per question.

Question	DK/NA	Totally Disagree	Partly Disagree	Partly Agree	Totally Agree
Q1	6.67%	0.00%	13.33%	66.67%	13.33%
Q2	6.67%	0.00%	13.33%	60.00%	20.00%
Q3	6.67%	0.00%	20.00%	46.67%	26.66%
Q4	33.33%	0.00%	13.33%	26.67%	26.67%
Q5	20.00%	0.00%	6.67%	53.33%	20.00%
Q6	20.00%	0.00%	13.33%	40.00%	26.67%
Q7	13.33%	0.00%	6.67%	46.67%	33.33%
Q8	6.67%	20.00%	0.00%	33.33%	40.00%
Q9	6.67%	0.00%	20.00%	46.67%	26.66%
Q10	26.66%	0.00%	6.67%	26.67%	40.00%
